# Synergistic interaction between hyperlipidemia and obesity as a risk factor for stress urinary incontinence in Americans

**DOI:** 10.1038/s41598-024-56744-5

**Published:** 2024-03-27

**Authors:** Fangyi Zhu, Mao Chen, Ya Xiao, Xiaoyu Huang, Liying Chen, Li Hong

**Affiliations:** Department of Gynaecology and Obstetrics, Renmin Hospital of Wuhan University, Wuhan, 430060 Hubei China

**Keywords:** Hyperlipidemia, Obesity, Stress urinary incontinence, Interaction, Biomarkers, Diseases, Endocrinology, Urology

## Abstract

Urinary incontinence is a common disease among middle-aged and elderly women, which not only affects the physical and mental health of patients, but also brings a great medical burden to society. Obesity is a known risk factor for urinary incontinence and is the most common secondary cause of hyperlipidemia. Most obese patients also suffer from hyperlipidemia in the clinic. However, few studies have explored the role of hyperlipidemia in women with urinary incontinence. Using data from the 2005–2018 National Health and Nutrition Examination Survey (NHANES), we aimed to evaluated the independent associations of high body mass index and hyperlipidemia with urinary incontinence in Americans by conducting a weighted multivariate logistic regression model. Additive interactions were also assessed using the relative excess risk due to interaction (RERI), attributed proportion of interaction (AP) and synergy index (S). This study demonstrated that hyperlipidemia was associated with a higher risk of stress urinary incontinence among women with obesity (OR = 1.52, 95% CI = 1.03–2.25), and there was a significant synergistic effect of hyperlipidemia and obesity on stress urinary incontinence(adjusted RERI: 3.75, 95% CI 0.30–7.20; adjusted AP: 0.67, 95% CI 0.54–0.80; adjusted S: 5.49, 95% CI 4.15–7.27). Moreover, fasting serum triglyceride lipids were the most relevant blood lipid indicator for the risk of stress urinary incontinence, especially among obese women younger than 50 years old, which contributes to the development of more refined lipid control protocols for patients with urinary incontinence in different age groups.

## Introduction

Urinary incontinence (UI) refers to a common disease in middle-aged and elderly women in which urine flows involuntarily from the external orifice of the urethra due to decreased or lost control of urination. UI can be divided into multiple types: stress urinary incontinence (SUI), urge urinary incontinence (UUI) and mixed urinary incontinence (MUI). The most common type is SUI, which is a condition in which involuntary urine leaks from the urethral orifice due to increased abdominal pressure from exertion, physical activity, sneezing, etc. Whereas UUI is defined as involuntary urine loss associated with a sense of urgency, MUI is a mix of the above symptoms^1^. Epidemiological studies confirm that the prevalence of any UI ranges from 25 to 45%, with more than 421 million people suffering from urinary incontinence worldwide, which is greater than the total population of the USA (329 million)^2,3^. Urinary incontinence reduces everyday competence and quality of life in all age groups. For example, urinary incontinence itself can lead to mobility inconvenience, which can lead to falls and mental impairments in elderly individuals. More importantly, because many women suffer in silence and accept UI as a normal part of the aging process, they may be underdiagnosed; however, even after the diagnosis, treatment and care are often inadequate^4^. In addition to affecting the physical and mental health of patients, UI also carries a significant economic burden, with the direct cost of incontinence-related care in the United States alone estimated at $19.5 billion^5^.

A body mass index (BMI) greater than 25 is considered to indicate overweight, and a BMI greater than 30 is considered to indicate obese. An imbalance in the capacity between caloric intake and expenditure can lead to obesity, which is regulated by multiple factors such as genetics, environment and individual behavior^6^. Global epidemiological surveys show a 28% increase in the prevalence of overweight and obesity in adults between 1980 and 2013, suggesting that nearly 2.1 billion people in the world are overweight or obese^7^. Several studies have suggested that obesity is linked to the development and severity of urinary incontinence, and weight loss should have a prominent place in treatment pathways for the management of UI^8,9^.

Hyperlipidemia is defined as a higher-than-normal level of one or more lipids in plasma, which is clinically classified as hypercholesterolemia, hypertriglyceridemia, mixed hyperlipidemia, and high-density lipoproteinemia in the clinic^10^. Epidemiological studies have confirmed that over 50% of American adults have elevated LDL levels, and the prevalence of dyslipidemia was significantly greater among white individuals than among Black individuals (women, 64.7% vs. 49.5%; and men, 78.4% vs. 56.7%; *P* < 0.001 for both)^11^.

In addition to being a known risk factor for urinary incontinence, obesity is also the most common secondary cause of hyperlipidemia, and most obese patients also suffer from hyperlipidemia in the clinic^12^. However, few groups have explored the role of hyperlipidemia in women with urinary incontinence, and people with hyperlipidemia without obesity or obesity without hyperlipidemia are often ignored in research on UI. Therefore, the objectives of this study were to assess the possible associations of hyperlipidemia and high BMI with urinary incontinence and the effect of the interaction between hyperlipidemia and obesity on stress urinary incontinence.

## Materials and methods

### Study population

The NHANES is a cross-sectional survey conducted by the National Center for Health Statistics to obtain a nationally representative sample of US noninstitutionalized residents through a multistage probability sample^13^. Since 1999, most data from this representative survey have been published online on a 2-year cycle, with participants first interviewed at home with a questionnaire and signing written informed consent. Then, they visit a screening center for a physical examination and laboratory tests^14^.

Due to a large amount of missing data after 2019 for COVID-19, we combined seven cycles of continuous NHANES data from 2005 to 2018 to include 34,043 female respondents for analysis. The National Center for Health Statistics Ethics Review Committee granted ethics approval, all procedures were performed in accordance with relevant guidelines. More information about the NHANES can be obtained at https://www.cdc.gov/nchs/nhanes/index.htm.

**Figure 1:**
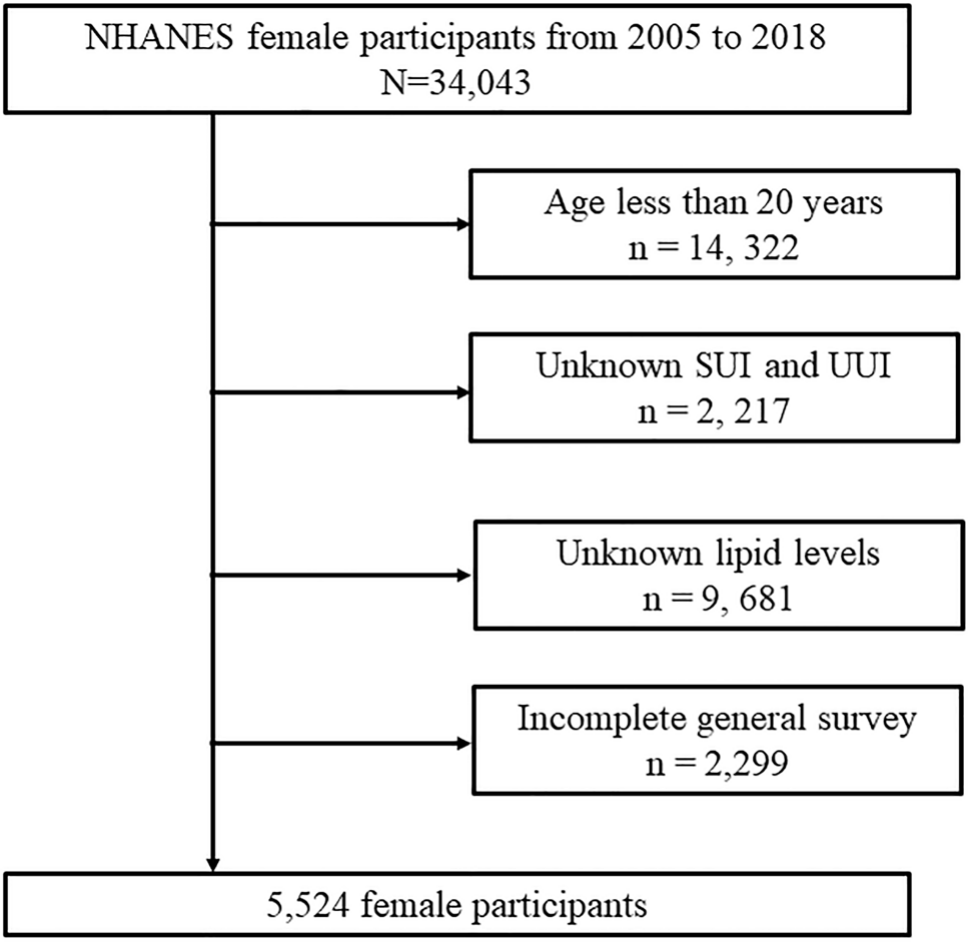
Flowchart of participant selection in this study. NHANES, National Health and Nutrition Examination Survey; SUI, stress urinary incontinence; UUI, urge urinary incontinence.

The exclusion criteria were as follows: (1) age less than 20 years (n = 14, 322); (2) unknown SUI and UUI (n = 2, 217); (3) unknown lipid levels (n = 9, 681); and (4) incomplete general survey (n = 2,299) (Fig. 1). As shown in Fig. 1, there are 34,043 female respondents included in seven cycles of continuous NHANES data from 2005 to 2018. Since urinary incontinence is a common disorder in middle-aged and elderly women in which urine flows involuntarily from the external orifice of the urethra due to decreased or lost control of urination, we first excluded respondents under 20 years of age by referring to the exclusion criteria of other published literature. Secondly, of the 19,721 female participants aged 20 years or older, 2217 had missing questionnaires related to urinary incontinence, 9681 had missing hematology laboratory values, and 2299 had missing data on confounding variables. After excluding these factors, 5,524 female participants were ultimately included in the final study.

### Outcome variable

The outcome of interest was the history of UI. This outcome was extracted from the Kidney Conditions—Urology file under Questionnaire Data. For the question “During the past 12 months, have you leaked or lost control of even a small amount of urine with an activity such as coughing, lifting, exercise, or an urge to urinate?”. Participants who answered “yes” were considered to have a history of SUI. The history of UUI was determined based on the question “During the past 12 months, have you leaked or lost control of even a small amount of urine with an urge or pressure to urinate and you could not get to the toilet fast enough?”. Participants who answered “yes” were considered to have a history of UUI. Mixed incontinence is defined as a condition that includes both stress and urge incontinence.

### Explanatory variables

NHANES organizers collected 3 ml or 5 ml of K3 EDTA anticoagulant whole blood from all Participants 18 years of age or older using established venipuncture protocols and procedures. Total cholesterol, triglycerides, serum low-density lipoprotein cholesterol (LDL-C), and serum high-density lipoprotein cholesterol (HDL-C) values were measured enzymatically. The diagnostic criteria for hyperlipidemia are as follows: 1. fasting serum triglycerides ≥ 150 mg/dl; 2. fasting serum total cholesterol ≥ 200 mg/dl; 3. HDL cholesterol < 50 mg/dl; 4. LDL cholesterol ≥ 130 mg/dl; meeting any of the above criteria was classified as hyperlipidemia. A BMI greater than 25 is considered to indicate overweight, and a BMI greater than 30 is considered to indicate obese.

### Confounding factors

All covariates were preselected based on known or suspected confounders of the relationship between hyperlipidemia and urinary incontinence and included age, race (non-Hispanic white, non-Hispanic black, Mexican American, other Hispanic, and other), number of vaginal deliveries (0, 1–2, ≥ 3), education (less than high school, high school or equivalent, college or above), hypertension (no, yes), income index (< = 1, 1–2, 2–5) and diabetes (no, yes). Diabetes was defined as the participant's self-reported diagnosis or hemoglobin A1c (HbA1c) ≥ 6.5% or both. Hypertension was defined as patients with three consecutive tests of systolic blood pressure (SBP) ≥ 140 mmHg or diastolic blood pressure (DBP) ≥ 90 mmHg. Statistical data files were merged with various data regarding demographics, laboratory tests, and questionnaires from 2005 to 2018 by the unique survey participant identifier SEQN. Considering the complex sampling method and the lack of inclusion of the largest proportion of lipid testing, we used sample weights specific to lipid testing data to generate national population estimates as directed in the NHANES reporting guidelines.

### Statistical analysis

Chi-square test was used to calculate the differences between categorical variables, while continuous variables were calculated using Student's t test. A weighted multivariate logistic regression model was used to assess the relationship between hyperlipidemia and various types of urinary incontinence, and the corresponding ORs and 95% CIs were calculated. The potential non-linear trend between hypertriglyceridemia and the prevalence of SUI was investigated by drawing a restricted cubic curve. The restricted cubic spline function is often used to explore nonlinear relationships between continuous variables and outcomes^15^. Biological interactions are divided into multiplicative and additive scales, where the additive scale better reflects the biological interactions^16^. Therefore, we coded the BMI(< 25 kg/m^2^, 25.0–29.9 kg/m^2^, > 30 kg/m^2^) and hypertriglyceridemia (no, yes) categories into six dummy variables and used the excel calculation table compiled by Andersson et al.^17^ to generate the estimated values of the relative excess risk due to interaction (RERI), the attributable proportion due to interaction (AP), the synergy index (SI), relative risk and 95% CI. When the RERI and AP intervals include 0 and the SI interval includes 1 indicates that the additive effect between risk factors is not statistically significant. All analyses were performed with R Studio software (version 1.2.4), and differences were considered statistically significant when *P* < 0.05.

Constrained cubic spline functions are powerful tools for exploring nonlinear relationships between continuous variables and outcomes, and can describe fairly well the dose–response relationship between the independent and dependent variables.

### Ethical approval

The studies involving human participants were reviewed and approved by National Center for Health Statistics (NCHS) research ethics review board. The patients/participants provided their written informed consent to participate in this study, all procedures were performed in accordance with relevant guidelines.

## Results

Table 1 shows the weighted prevalence (percentage) and 95% CIs of confounding factors in U.S. women included in the NHANES 2005–2018 survey. The mean age (95% CI) of all women was 52.0 (51.4–52.6) years, with approximately 69.4% reporting non-Hispanic white race. Approximately 40.0% of participants were obese in both age subgroups. Other confounding factors related to urinary incontinence all varied (significantly) by age group (20–49 years vs. 50 to > 85 years). We found a higher proportion of participants in women 50 and older who were non-Hispanic white (75.7% vs. 61.6%, 69.4%), had diabetes (25.3% vs. 7.9%, 17.5%), had a high income index (66.8% vs. 58.1%, 62.9%), and had a university degree and above (63.9% vs. 59.5%, 56.0%) than in young women. The number of vaginal deliveries in women 50 and older also tended to be higher than that in young women.

**Table1:** Weighted prevalence and 95% confidence intervals of confounding factors in the US. Women stratified by age.

Variable	Variable*Category	Ages 20–49 (n = 2299)	Ages 50 to older than 85 (n = 3225)	Ages 20 to older than 85 (n = 5524)	*P* value
Race^†^					< 0.0001
Non-Hispanic White	2453	61.57 (57.92, 65.22)	75.67 (73.05, 78.29)	69.40 (63.30, 75.50)	
Non-Hispanic Black	1151	13.41 (11.37, 15.45)	10.16 (8.49, 11.83)	11.60 (10.15, 13.05)	
Mexican American	835	10.95 (9.07, 12.83)	4.52 (3.51, 5.52)	7.37 (6.20, 8.55)	
Other Hispanic	570	6.32 (5.02, 7.62)	3.94 (3.07, 4.82)	5.00 ( 4.12, 5.87)	
Other	515	7.76 (6.28, 9.23)	5.72 (4.63, 6.81)	6.63 ( 5.65, 7.60)	
Education^†^					< 0.0001
Less than high school	2092	30.54 (28.19, 32.89)	28.27 (25.34, 31.20)	32.38 (30.11, 34.64)	
High school or equivalent	560	9.98 ( 8.52, 11.44)	7.88 ( 6.52, 9.24)	11.67 (10.12, 13.22)	
College or above	2872	59.45 (55.26, 63.63)	63.85 (60.62, 67.08)	55.95 (53.60, 58.30)	
Income index^†^					< 0.0001
< = 1	1534	20.47 (18.32, 22.63)	11.47 ( 9.80, 13.13)	15.47 (13.98, 16.96)	
1–2	1267	21.43 (19.48, 23.39)	21.77 (19.87, 23.66)	21.62 (19.91, 23.32)	
2–5	2723	58.10 (55.05, 61.14)	66.77 (64.16, 69.37)	62.91 (58.14, 67.69)	
Hypertension^†^					< 0.0001
No	2890	78.96 (76.87, 81.05)	39.48 (37.14, 41.82)	57.02 (53.32, 60.73)	
Yes	2634	21.04 (18.95, 23.13)	60.52 (58.18, 62.86)	42.98 (40.10, 45.85)	
Vaginal deliveries^†^					< 0.0001
0	1009	26.82 (24.85, 28.79)	13.97 (11.95, 16.00)	19.68 (17.91, 21.46)	
1–2	2359	48.99 (46.23, 51.75)	45.13 (42.87, 47.39)	46.84 (43.65, 50.04)	
> = 3	2156	24.19 (21.74, 26.64)	40.90 (38.58, 43.21)	33.47 (30.81, 36.14)	
Diabetes^†^					< 0.0001
No	4275	78.86 (76.48, 81.24)	53.95 (51.40, 56.50)	65.02 (60.78, 69.26)	
Yes	1249	7.86 ( 6.42, 9.30)	25.27 (23.23, 27.32)	17.54 (16.02, 19.06)	
BMI^†^					
Underweight	86	1.95 (1.27, 2.63)	1.48 (1.05, 1.91)	1.69 ( 1.28, 2.09)	0.002
Normal	1430	31.37 (28.52, 34.21)	26.26 (24.17, 28.34)	28.53 (26.02, 31.03)	
Overweight	1615	26.37 (24.38, 28.35)	30.81 (28.54, 33.08)	28.84 (26.67, 31.00)	
Obese	2393	40.32 (37.78, 42.86)	41.46 (38.94, 43.98)	40.95 (37.90, 44.01)	

*BMI* body mass index.

Data are % (95% confidence interval) or n.

*For the total group of women, ages 20 years and older.

^†^*P* < 0.05 from Rao-Scott adjusted χ2; comparison of each confounding factor across age groups.

**Figure 2:**
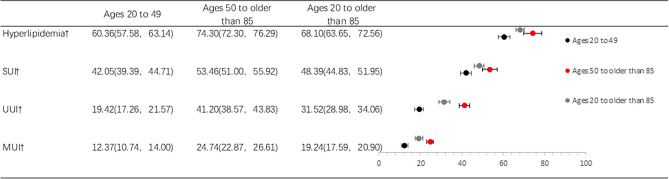
Weighted prevalence and 95% confidence intervals of hyperlipidemia and urinary incontinence in U.S. women stratified by age. SUI, stress urinary incontinence; UUI, urge urinary incontinence; MUI, mixed urinary incontinence. Data are % (95% confidence interval). ^†^*P* < 0.05 from Rao-Scott adjusted χ2; comparison of hyperlipidemia and urinary incontinence across age groups.

Figure 2 summarizes the weighted frequencies of hyperlipidemia and various types of urinary incontinence for different age groups and for the total sample. Overall, as the most common form of urinary incontinence, the weighted prevalence of stress urinary incontinence was 48.4%, while the weighted prevalence of urgent urinary incontinence and mixed urinary incontinence were 31.5% and 19.2%, respectively. A total of 68.1% of women were reported to have hyperlipidemia. More importantly, the prevalence of hyperlipidemia and urinary incontinence that increased with age was significantly different between the two age groups.

Table 2 shows the weighted prevalence of confounding factors and three types of urinary incontinence by hyperlipidemia in women stratified by age. In the group under 50 years old, income index and education showed an inverse association with prevalence of hyperlipidemia; those with low income index as well as education level are prone to hyperlipidemia. Consistent with expectations, the prevalence of the three types of urinary incontinence was significantly higher in individuals with hyperlipidemia than in those without hyperlipidemia. However, for women over 50 years of age, only the prevalence of stress urinary incontinence is not significantly increased in women with hyperlipidemia among the three types of urinary incontinence, and non-Hispanic white individuals are more likely to suffer from hyperlipidemia relative to non-Hispanic African Americans and Mexican Americans. Obesity were more frequent in women with hyperlipidemia in both age subgroups.

**Table 2:** Weighted prevalence and 95% confidence intervals of confounding factors and three types of urinary incontinence by hyperlipidemia in U.S. women stratified by age.

	Ages 20–49			Ages 50 to older than 85		
Variable	Normal	Hyperlipidemia	*P* value	Normal	Hyperlipidemia	*P* value
Race^†^			0.07			0.002
Non-Hispanic White	61.25 (56.59, 65.91)	61.77 (57.80, 65.75)		73.74 (69.66, 77.83)	76.33 (73.68, 78.98)	
Non-Hispanic Black	15.16 (12.17, 18.15)	12.26 (10.15, 14.36)		13.16 (10.22, 16.11)	9.12 ( 7.60, 10.63)	
Mexican American	8.98 ( 6.61, 11.35)	12.24 (10.16, 14.33)		4.42 (3.13, 5.71)	4.55 (3.53, 5.57)	
Other Hispanic	6.44 (4.78, 8.10)	6.24 (4.65, 7.82)		2.66 (1.83, 3.48)	4.39 (3.36, 5.42)	
Other	8.17 (6.07, 10.26)	7.49 (5.68, 9.30)		6.02 (4.23, 7.81)	5.62 (4.31, 6.93)	
Education*			< 0.001			0.96
College or above	69.49 (64.92, 74.05)	60.15 (56.74, 63.57)		56.38 (52.01, 60.75)	55.80 (52.92, 58.69)	
High school or equivalent	6.60 (4.64, 8.56)	8.71 (7.01, 10.42)		11.65 (9.10, 14.19)	11.68 (9.99, 13.37)	
Less than high school	23.91 (19.95, 27.87)	31.13 (27.86, 34.41)		31.97 (27.77, 36.18)	32.52 (29.82, 35.21)	
Income index*			0.03			0.43
1–2	18.57 (15.54, 21.61)	23.31 (20.62, 26.01)		20.83 (18.11, 23.55)	22.09 (19.87, 24.31)	
< = 1	19.21 (15.97, 22.45)	21.30 (18.58, 24.02)		12.74 (9.66, 15.82)	11.03 (9.32, 12.74)	
2–5	62.22 (57.69, 66.74)	55.39 (52.06, 58.72)		66.43 (62.33, 70.53)	66.88 (64.08, 69.68)	
Vaginal deliveries			0.26			0.06
0	25.08 (21.97, 28.19)	27.96 (25.07, 30.86)		11.70 ( 8.63, 14.78)	14.76 (12.27, 17.25)	
1–2	51.52 (47.05, 55.98)	47.33 (43.88, 50.78)		42.23 (36.83, 47.62)	46.13 (43.55, 48.72)	
> = 3	23.40 (19.82, 26.98)	24.71 (21.88, 27.54)		46.07 (40.69, 51.45)	39.11 (36.46, 41.76)	
Hypertension*^†^			< 0.0001			0.04
No	86.63 (84.20, 89.05)	73.99 (71.03, 76.95)		35.65 (31.37, 39.94)	41.28 (38.31, 44.24)	
Yes	13.37 (10.95, 15.80)	26.01 (23.05, 28.97)		64.35 (60.06, 68.63)	58.72 (55.76, 61.69)	
Diabetes*			< 0.0001			0.79
No	97.36 (96.41, 98.31)	88.71 (86.50, 90.92)		75.14 (71.42, 78.85)	74.58 (72.31, 76.86)	
Yes	2.64 (1.69, 3.59)	11.29 (9.08, 13.50)		24.86 (21.15, 28.58)	25.42 (23.14, 27.69)	
BMI*^†^			< 0.0001			0.03
Normal	48.02 (43.84, 52.21)	20.43 (17.25, 23.60)		31.13 (26.65, 35.61)	24.57 (22.00, 27.14)	
Underweight	3.68 (2.17, 5.19)	0.81 (0.26, 1.36)		2.00 (0.83, 3.16)	1.30 (0.83, 1.76)	
Overweight	24.69 (21.53, 27.84)	27.47 (24.76, 30.18)		29.09 (25.03, 33.14)	31.41 (28.88, 33.93)	
Obese	23.61 (20.33, 26.89)	51.29 (48.17, 54.41)		37.79 (33.45, 42.13)	42.73 (39.93, 45.52)	
SUI*			0.02			0.65
No	62.23 (57.98, 66.48)	55.13 (51.49, 58.77)		47.41 (43.42, 51.39)	46.24 (43.22, 49.27)	
Yes	37.77 (33.52, 42.02)	44.87 (41.23, 48.51)		52.59 (48.61, 56.58)	53.76 (50.73, 56.78)	
UUI*^†^			< 0.001			0.004
No	85.37 (82.44, 88.30)	77.44 (74.37, 80.51)		52.89 (48.56, 57.22)	60.84 (57.67, 64.01)	
Yes	14.63 (11.70, 17.56)	22.56 (19.49, 25.63)		47.11 (42.78, 51.44)	39.16 (35.99, 42.33)	
MUI*^†^			0.002			0.03
No	91.05 (88.72, 93.39)	85.38 (83.14, 87.63)		71.55 (67.89, 75.22)	76.54 (74.25, 78.84)	
Yes	8.95 ( 6.61, 11.28)	14.62 (12.37, 16.86)		28.45 (24.78, 32.11)	23.46 (21.16, 25.75)	

*SUI* stress urinary incontinence, *UUI* urge urinary incontinence, *MUI* mixed urinary incontinence, *BMI* body mass index.

Data are % (95% confidence interval) or n. *P* values from a Rao-Scott adjusted χ2.

*P < 0.05 in comparison of confounding/outcome variables by hyperlipidemia in women aged 20 to 49.

^†^*P* < 0.05 in comparison of confounding/outcome variables by hyperlipidemia in women aged 50 years and older.

**Figure 3:**
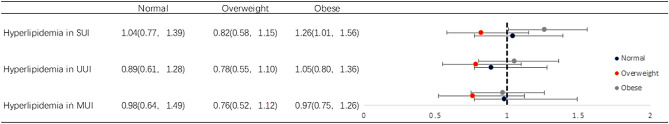
Multivariable logistic regression models summarizing the association between hyperlipidemia and urinary incontinence in U.S. Women at differe nt body mass index levels. Data are adjusted odds ratios (95% confidence intervals). *Adjusted covariables: age (continuous variable), race (four categories), number of vaginal deliveries (three categories), education (three categories), hypertension (two categories), income index (three categories) and diabetes (two categories).

Obesity is the most common secondary cause of hyperlipidemia. In the clinic, most obese patients also suffer from hyperlipidemia^12^. Figure 3 shows the results of a multivariable weighted logistic regression analysis between hyperlipidemia and urinary incontinence in U.S. Women at different body mass index levels. The adjusted OR (95% CI) was 1.26 (1.01, 1.56) in the obese population, indicating that compared with women without hyperlipidemia, the risk of stress urinary incontinence in obese women with hyperlipidemia significantly increased by approximately 26.0%. In contrast, the incidence of stress urinary incontinence in patients with hyperlipidemia was not statistically significant in those with body mass index < 25 kg/m^2^ (OR: 1.04; 95% CI 0.77, 1.39) and those with overweight (OR: 0.82; 95% CI 0.58, 1.15). There was also no significant correlation between hyperlipidemia and urgent urinary incontinence or mixed urinary incontinence in women at different body mass index levels.

Table 3 shows the results of a multivariable weighted logistic regression analysis after adjusting for age, race, education, income index, vaginal delivery, diabetes, hypertension and body mass index to assess whether hyperlipidemia has an impact on stress urinary incontinence in obese women stratified by age. Only in obese women under 50 years of age was hyperlipidemia positively correlated with the risk of stress urinary incontinence [adjusted OR (95% CI) was 1.52 (1.03,2.25), *P* = 0.04], while in obese women over 50 years of age, the correlation between hyperlipidemia and stress urinary incontinence was not statistically significant [adjusted OR (95% CI) was 1.09 (0.81,1.48), *P* = 0.56]. After further analysis of various blood lipid indicators, it was found that fasting serum triglyceride lipids were the most relevant blood lipid indicator for the risk of SUI. Especially in women under 50 years of age, hypertriglyceridemia is positively correlated with the risk of stress urinary incontinence [adjusted OR (95% CI) is 1.95 (1.35,2.82), *P* =  < 0.001], which shows that young obese women are more vulnerable to the effects of hypertriglyceridemia, which significantly increases the risk of stress urinary incontinence by approximately 95%.

**Table 3:** Multivariable logistic regression model summarizing the association between blood lipid indicators and stress urinary incontinence in U.S. obese women Stratified by Age.

	Ages 20–49 (n = 2299)		Ages 50 to older than 85 (n = 3225)	
Dependent variable	OR (95% CI)	Pr (>|t|)	OR (95% CI)	Pr (>|t|)
Hyperlipidemia				
No	Reference	0.04	Reference	0.56
Yes	1.52 (1.03, 2.25)		1.09 (0.81, 1.48)	
Fasting serum triglycerides, mg/dl				
< 150	Reference	< 0.001	Reference	0.05
> = 150	1.95 (1.35, 2.82)		1.41 (1.00, 1.99)	
Fasting serum total cholesterol, mg/dl				
< 200	Reference	0.05	Reference	0.79
> = 200	1.40 (1.01, 1.94)		0.96 (0.69, 1.32)	
High-density lipoprotein cholesterol, mg/dl				
> = 50	Reference	0.11	Reference	0.40
< 50	1.32 (0.93, 1.85)		1.16 (0.82, 1.63)	
Low-density lipoprotein cholesterol, mg/dl				
< 130	Reference	0.15	Reference	0.48
> = 130	1.25 (0.92, 1.70)		1.12 (0.81, 1.55)	

Adjusted covariables: age (continuous variable), race (four categories), number of vaginal deliveries (three categories), education (three categories), hypertension (two categories), income index (three categories), diabetes (two categories), body mass index (continuous variable).

**Figure 4:**
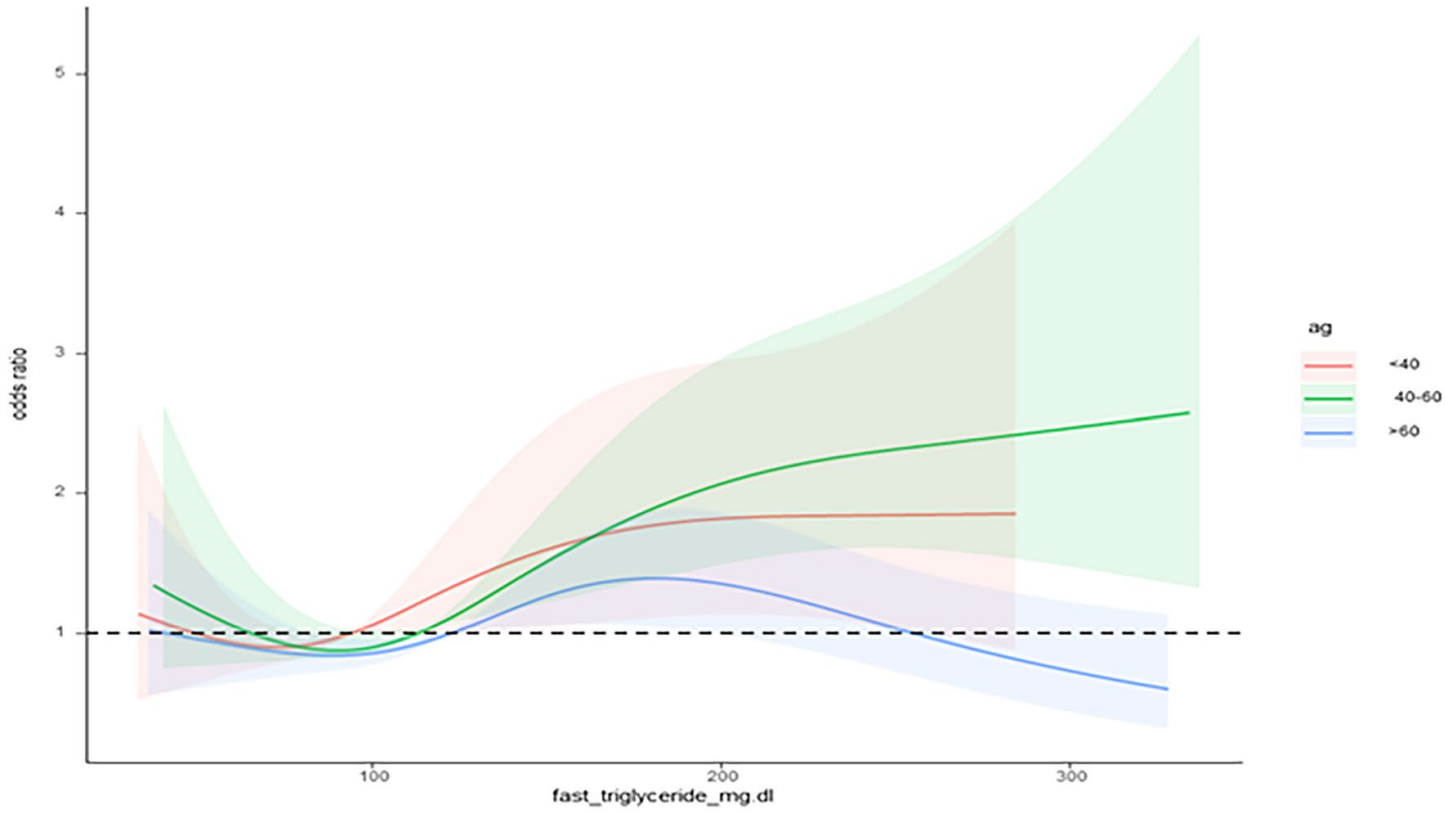
Restricted cubic spline model images of blood glucose and stress urinary incontinence (SUI) prevalence. *Adjusted covariables: age(continuous variable), race (four categories), number of vaginal deliveries (three categories), education (three categories), hypertension (two categories), income index(three categories), diabetes(two categories), body mass index(continuous variable).

We also established a restricted cubic spline model after adjusting for age, race, education, income index, vaginal delivery, diabetes, hypertension and body mass index in obese women stratified by age. In women aged 20–40 years old, there was a nonlinear positive correlation between fasting serum triglyceride lipid levels and the prevalence of SUI, with an inflection point (a change in the dose‒response relationship) of approximately 95 mg/dl. In women aged 40–60 years old, the prevalence of SUI increased most significantly as fasting serum triglyceride lipids increased, with an inflection point (a change in the dose‒response relationship) of approximately 110 mg/dl. In women over 60 years old, the prevalence of SUI did not change significantly with the level of fasting serum triglyceride lipids (Fig. 4).

**Figure 5:**
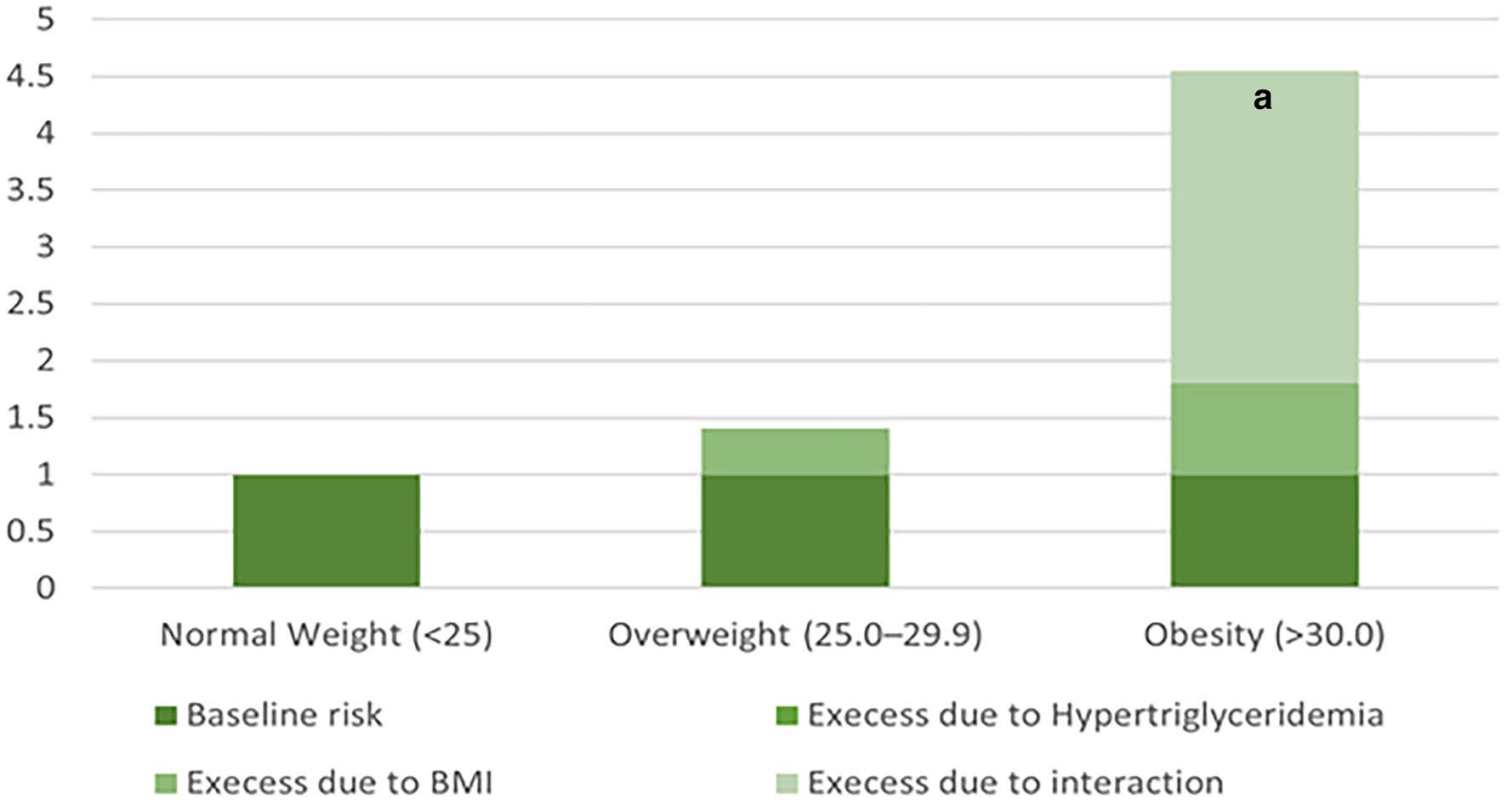
Synergistic interaction effect between hypertriglyceridemia and high BMI on stress urinary incontinence. ^a^*P* < 0.05 and indicated a synergistic interaction by synergy index.

Table 4 shows the independent effect and additive interaction of hypertriglyceridemia and high BMI on stress urinary incontinence. Patients with a BMI < 25 kg/m^2^ without hypertriglyceridemia were used as controls (OR = 1). The OR value of obesity (BMI >  = 30 kg/m^2^) alone on stress urinary incontinence was increased from 1.80 (95% CI 1.45–2.25) in those without hypertriglyceridemia to 3.00 (95% CI 2.24–4.01) in those with hypertriglyceridemia, and an additive interaction was found between hypertriglyceridemia and obesity in stress urinary incontinence risk (adjusted RERI: 3.75, 95% CI 0.30–7.20; adjusted AP: 0.67, 95% CI 0.54–0.80; adjusted S: 5.49, 95% CI 4.15–7.27). In contrast, there was no significant synergistic interaction between hypertriglyceridemia and overweight (BMI 25.0–29.9 kg/m^2^) for the risk of stress urinary incontinence. Figure 5 also shows the statistical analysis of the additive interaction to support the results. The contribution of the additive interaction varied for different BMI groups, with the attributable proportion (AP) significantly higher in the obesity group than in the overweight group, 0.67 (95% CI 0.54–0.80) versus 0.14 (95% CI − 0.18–0.46), which indicates that 67% of cases of stress urinary incontinence were caused by the interaction between hypertriglyceridemia and obesity in the samples of this study.

**Table 4:** Interactive effect analysis of hypertriglyceridemia and high BMI in U.S. women.

		Normal Weight (< 25)		Overweight (25.0–29.9)		Obesity (> 30.0)	
Hypertriglyceridemia	Hign BMI	OR (95% CI)	Pr (>|t|)	OR (95% CI)	Pr (>|t|)	OR (95% CI)	Pr (>|t|)
0	0	Reference					
1	0	1.03 (0.65, 1.65)	0.89				
0	1			1.41 (1.09, 1.82)	0.01	1.80 (1.45, 2.25)	< 0.0001
1	1			1.15 (0.84, 1.60)	0.38	3.00 (2.24, 4.01)	< 0.0001
	RERI (95% CI)	Reference		0.23 (-0.46, 0.92)		3.75 (0.30, 7.20)	
	AP (96% CI)	Reference		0.14 (-0.18, 0.46)		0.67 (0.54, 0.80)	
	S (97% CI)	Reference		1.53 (0.72, 3.26)		5.49 (4.15, 7.27)	

Adjusted covariables: age (continuous variable), race (four categories), number of vaginal deliveries (three categories), education (three categories), hypertension (two categories), income index (three categories) and diabetes (two categories). *BMI* body mass index, *RERI* relative excess risk due to interaction, *AP* attributable proportion due to interaction, *SI* synergy index.

## Discussion

In the present study, we found that individuals with hyperlipidemia had a significantly higher odds ratio for stress urinary incontinence in women with obesity, and fasting serum triglyceride lipids were the most relevant blood lipid indicator for the risk of stress urinary incontinence. Furthermore, the dose‒response curves showed a nonlinear positive correlation between fasting serum triglyceride lipids and the prevalence of SUI in women under 60 years of age with obesity, and there was a significant synergistic interaction between hypertriglyceridemia and obesity on stress urinary incontinence, which suggests that elevated fasting serum triglyceride lipid levels may be a strong biomarker for SUI in women with obesity.

In addition to age and vaginal delivery, obesity is also an important risk factor for urinary incontinence^18^. The effect of obesity on SUI is not only due to increased intra-abdominal pressure, but may also be involved in SUI-related alterations in neurological aspects. Some studies have confirmed that the effect of obesity on neurological function may be exacerbated by the coexisting metabolic conditions possibly by causing inflammation, oxidative stress, impaired blood vessel formation, hormonal disruption, which may explain the additive effect of hypertriglyceridemia and obesity on the risk of stress urinary incontinence^19^.

Metabolic diseases such as obesity are often accompanied by hyperlipidemia^20^. Epidemiological studies have shown that hyperlipidemia is a risk factor for white matter hyperintensities and overactive bladder syndrome and that these disorders can lead to urinary incontinence^21,22^. Animal experiments confirmed that hyperlipidemic mice showed a significant loss of urethral spontaneous tone compared to the normal group^23^. Additionally, evidence suggests that the onset of neurodegeneration occurs early in the disease, including activation of inflammatory pathways, reduction of neuroprotective factors, DNA damage, and apoptosis. Oxidative stress amplifies these processes and is elevated in the setting of hyperlipidemia^24^. Huali Wu et al. also confirmed that simvastatin (a type of lipid-lowering drug) can reduce neuronal damage induced by hyperlipidemia^25^. Therefore, we speculate that hyperlipidemia may damage the peripheral nerve structure of the pelvic floor by inducing oxidative stress and then participate in the pathogenesis of stress urinary incontinence.

Urinary incontinence increases in prevalence with age, and the increasing mean life expectancy in the world will lead to an expected increase in the number of people with UI in the future. Another important factor to consider is that women with a higher social status have higher expectations for quality of care. Thus, the demands on health-care services regarding the management of UI are expected to increase in the future, due in part to the aging population^3,26^. Therefore, it is necessary to develop more sophisticated prevention and treatment strategies for urinary incontinence.

Our findings contribute to further understanding of the relationship between hyperlipidemia and stress urinary incontinence, and emphasize the importance of controlling lipid levels to reduce the risk of stress urinary incontinence in obese women. More importantly, the findings of the restricted cubic spline model provide a more nuanced understanding of how hyperlipidemia is associated with stress urinary incontinence by age, which can help inform future hyperlipidemia guidelines and interventions that target obese women in different age groups differently. The substantial differences seen in associations for obese women in different age groups should be considered when setting hyperlipidemia goals and monitoring public health progress to reduce the risk of stress urinary incontinence. Among the inflection points identified for women between the ages of 40 and 60, fasting serum triglycerides should be controlled at less than 110 kg/m^2^, rather than the more stringent standard of less than 95 kg/m^2^ triglycerides in women under 40 years of age. The fasting serum triglyceride control index was more relaxed in women over 60 years of age than in younger women. The enhanced understanding of the association between hyperlipidemia and stress urinary incontinence in obese women underscores the importance of continued work to control blood lipid levels to reduce the risk of stress urinary incontinence in obese women.

This study also has several limitations. First, there was potential selection bias due to missing visits. However, 39% of young women were aged 40 years and younger, 31% of middle-aged women were aged 40–59 years, and 30% of older women were aged 60 years and older, so we assume that the missing data were evenly distributed between age groups so that the prevalence between groups may not be affected. Second, all information was obtained through self-report. Therefore, information bias may affect the accuracy of the data. Additionally, our study did not include diet and medication use in the comparative analysis, and they may have influenced the lipid and study results.

### Supplementary Information

Supplementary Information.

## Data Availability

The datasets generated and analysed during the current study are available in the [National Health and Nutrition Examination Surveys] repository, this data can be found here: NHANES—National Health and Nutrition Examination Survey Homepage (cdc.gov).
